# Illustration of the variable 1D sequences but conserved 2D and 3D structures of different ncRNA nanostructures for tracking the evolution and origin of organisms

**DOI:** 10.1016/j.ncrna.2025.09.003

**Published:** 2025-09-30

**Authors:** Kai Jin, Margaret Bohmer, Peixuan Guo

**Affiliations:** a Division of Pharmaceutics and Pharmacology, College of Pharmacy, The Ohio State University, Columbus, OH, 43210, USA; b Center for RNA Biology, The Ohio State University, Columbus, OH, 43210, USA; c Center for RNA Nanobiotechnology and Nanomedicine, College of Medicine, Dorothy M. Davis Heart and Lung Research Institute, and Comprehensive Cancer Center, The Ohio State University, Columbus, OH, 43210, USA

**Keywords:** RNA hexamer, Biomotor structure, Viral machine, Nanobiotechnology, DNA-packaging, Viral assembly, Computational Biology

## Abstract

Viruses and other microorganisms are constantly mutating and emerging in different regions. Here, we apply nanotechnology to examine the primary, secondary, and tertiary structures of novel noncoding RNA nanoparticles and find that primary sequences (1D) vary widely between viruses, while secondary (2D) and tertiary structures (3D) are highly conserved. The uniqueness of the phi29 system makes this reported study possible. RNA evolution studies are relevant to the concept of RNA as the origin of life. The recent discovery that RNA is a motile and recombinant entity supports the hypothesis that RNA is the substance of life. Previously, we reported noncoding RNA nanoparticles packaging RNA (pRNA) of the Bacillus virus phi29 (Nature Nanotechnology, 2011, PMID: 21909084; Nature Nanotechnology, 2010, PMID: 21102465; Science, 1987, PMID: 3107124). Phi29 can infect spore-forming Bacillus subtilis, which has long been hidden in the soil with slow or no evolution due to the protection of spores. They are living fossils. The pRNA of the phi29 DNA packaging motor drives the viral motor for DNA transport. In this study, we used existing datasets to search for more pRNAs. Their primary sequence diversity makes it challenging to identify them from the whole genomes of other species. Using the top-down (1D) approach and the bottom-up assembly (3D) approaches, we found that their 2D and 3D structures are highly conserved. Structural conservation enabled us to apply the two-dimensional structure-based approach to find these ncRNAs from databases and identify 12 new pRNAs. The presence of two additional components in the genome, a motor channel protein and a motor ATPase, further confirmed the authenticity of these pRNAs and supported the conclusion that these pRNAs are motor-driven components. Highly conserved and paired left and right loops for assembling the pRNA hexamer were identified in all 12 pRNAs. Artificial modification of the pairing and determination of the virion production activity of the mutated phi29 pRNA further confirmed the conclusion that the secondary and tertiary structures are highly conserved. Understanding the retention, conservation, and variation of viral non-coding RNA sequences and structure can help us trace the evolutionary history of the virus, find lineage information, and provide important information about the origin of the viruses. It can also provide knowledge for the design of disease prevention and treatment by providing the background for *in vivo* RNA nanotechnology.

## 1 Introduction

According to the “RNA world” hypothesis, RNA may be the start of life due to its ability to both store genetic information and catalyze chemical reactions [[1], [2], [3], [4]]. Recent discoveries about RNA adaptability, stability, and motility further support this hypothesis, indicating that RNA may have played a central role in the early evolutionary history of species [2,[5], [6], [7], [8], [9], [10]].

Human genome sequencing revealed that only ∼1.5 % of the chromosomal dsDNA sequence codes for proteins. Subsequent studies found that much of the 98.5 % so‐called “junk” DNAs code for noncoding RNAs (ncRNAs), which are RNA transcripts that do not undergo translation to form proteins [11]. Further studies have demonstrated that a significant portion of this non-coding DNA is involved in the production transcription of ncRNAs [12]. ncRNAs are not translated into proteins; however, ncRNAs perform essential regulatory and structural roles in gene expression and other cellular processes [13,14], such as RNA interference (RNAi) [[15], [16], [17], [18]]. Small non-coding RNAs range in size from 15 to 200 nucleotides and play important roles in functional regulation in many species. Examples of small ncRNAs include small interfering RNAs (siRNAs) [[19], [20], [21]], microRNAs (miRNAs) [22], Piwi-interacting RNAs (piRNAs) [23], and more. Long non-coding RNAs (lncRNAs) with sizes longer than 200 nucleotides have also gained attention for their functions in gene regulation, chromatin remodeling, and scaffolds for protein complexes [[24], [25], [26]].

Packaging RNA (pRNA) is an example of small non-coding RNA functionalized in the bacteria virus phi29 DNA packaging motor. These pRNAs are important for the assembly and function of the DNA packaging motor in phi29 [27,28]. Furthermore, it was previously found that the phi29 pRNA can bind ATP due to its similarity to the synthetic Sassanfar-Szostak ATP aptamer, including a conserved G bulge [29]. Herein, we speculate that pRNA may widely exist in nature, forming as part of the DNA packaging motors in other viruses. This study aims to expand the understanding of pRNA and explore the prevalence of pRNA in natural datasets by utilizing several computational methods.

By screening through extensive sequencing data of different viruses, pRNAs with a significant homology and structural conservation were identified; some of them were not previously linked to packaging function. It was further revealed that these identified pRNAs are present in viral genomes that encode essential protein components of DNA packaging. Such proteins include ones that are homologous to phi29 ATPase gp16 and connector gp10. The existence of these functional proteins strongly supports the idea that these pRNAs belong to a secondary structure-conserved class of dsDNA-packaging motor-associated RNAs. Furthermore, pRNA sequences not only exist in the Salasvirus genus but also currently unclassified bacteria viruses such as *Cytobacillus* phage Bfsp1, indicating a wide taxonomic distribution of pRNAs.

The identification methods, detailed sequences, and hexamer-forming structures of several high-confidence newly found pRNAs are reported here, suggesting that packaging motor-related pRNAs are indeed widespread in nature. The secondary and tertiary structures are highly conserved as verified by using Infernal and AlphaFold, while the primary sequences of found pRNAs are diverse. These findings may contribute to the research on the function of non-coding RNAs, and further our understanding of RNA's evolutionary and functional roles within different biological contexts.

## 2 Results

### 2.1 Identification of 12 pRNAs from the whole virus database by 2D structure prediction program, not primary sequence search

**Fig. 1:**
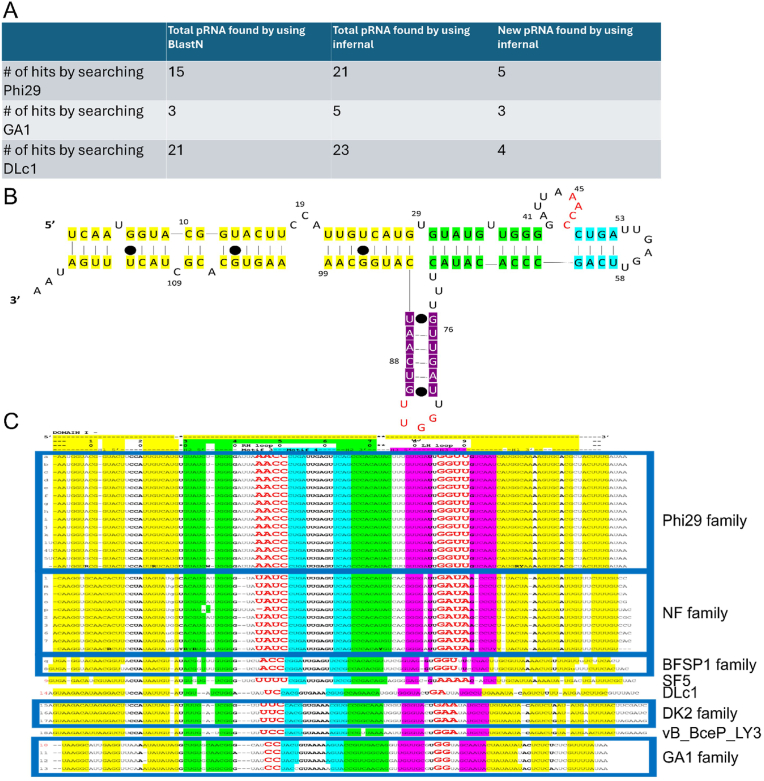
Secondary structure alignment of a variety of pRNA species found in genome database. The computer programs of BlastN and Infernal were used. pRNA sequences of Phi29, GA1, and Dlc1 were used as templates for the search. **(A)** Outcome of the search. **(B)** The schema of Phi29 pRNA folding showing the indicating the color of each section in consistent with panel C **(C)** Primary sequence alignment of the resulting pRNAs categorized based on the sequences of the Right-hand Loop (RH) and Left-hand Loop (LH) into eight different groups.

**Fig. 2:**
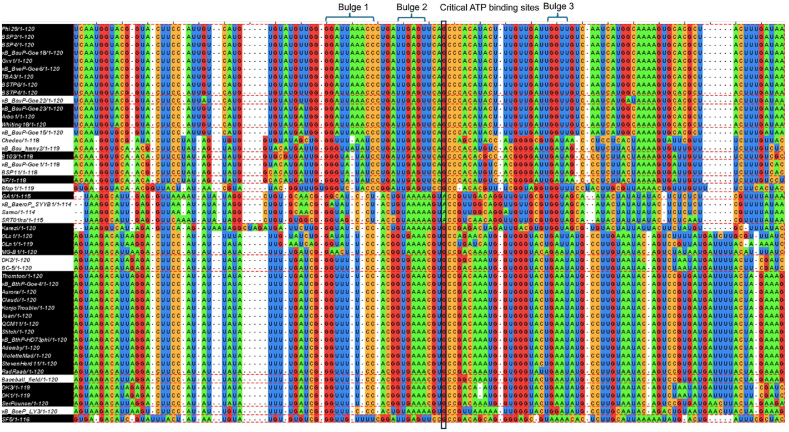
Primary sequence alignment of pRNAs via Jalview. the black background pRNAs are previously reported, and the plain background pRNAs are newly identified.

BlastN is a homologous search program based on primary sequences [30], but primary sequence similarity searches from the database could not identify all pRNAs (Fig. 1A). This might be due to the heterogeneity and diversity of the primary sequence of pRNAs, as shown in Fig. 2. Phi29, GA1, or DLc1 pRNA domain 1 were used as the seed, and BlastN searching cannot get the same result compared to searching via Infernal (Fig. 1A). Infernal was able to identify more pRNAs compared to BlastN.

Infernal is a tool for searching for RNA with homologous 2D structures [31]. Herein, the motor pRNAs of phi29, GA1, and DLc1 pRNA domain 1 were used as templates to search for new pRNAs using the Infernal. The parameters of E-value < e−6 and sequence coverage > 0.8 were used as criteria to identify new sequences. Search results generated 21 total hits from phi29, five total hits from GA1, and 25 total hits from DLc1 (Fig. 1A). Furthermore, we previously identified B103, SF5, and NF pRNAs [32], and the pRNA domain 1 sequences of B103, SF5, and NF are also included in the results to illustrate a complete list of found pRNAs. Jalview was used to align pRNA hits found via Infernal, and the alignment of four important functional sections (Bulge1, Bulge2, Critical ATP-binding sites, and Bulge3) of pRNAs are listed in Fig. 1, Fig. 2.

Within these 50 hits, 16 from phi29, two from GA1, 19 from DLc1, and SF5 from our previous paper [32] have been previously published in the literature. Thus, 12 pRNAs were identified as new findings (Fig. 1A). Domain 1 of 12 pRNAs, along with 6 source pRNAs and other duplicated pRNAs, were further aligned as shown in Fig. 1, Fig. 2. Self-folding paradigm is further shown in Fig. 1, Fig. 2B–C with helix and bulge structures identified, and the four important functional sections (Bulge 1, 2, and 3 and Critical G) were listed in Fig. 2.

### 2.2 The presence of the paired right-hand loop (RH) with the left-hand loop (LH) and their strong covariation

It is very notable that all pRNA species contained the paired right-hand loop (RH) and the left-hand loop (LH). The sequences of RH and LH are not identical, but all are nicely paired via three to four nucleotides.

**Fig. 3:**
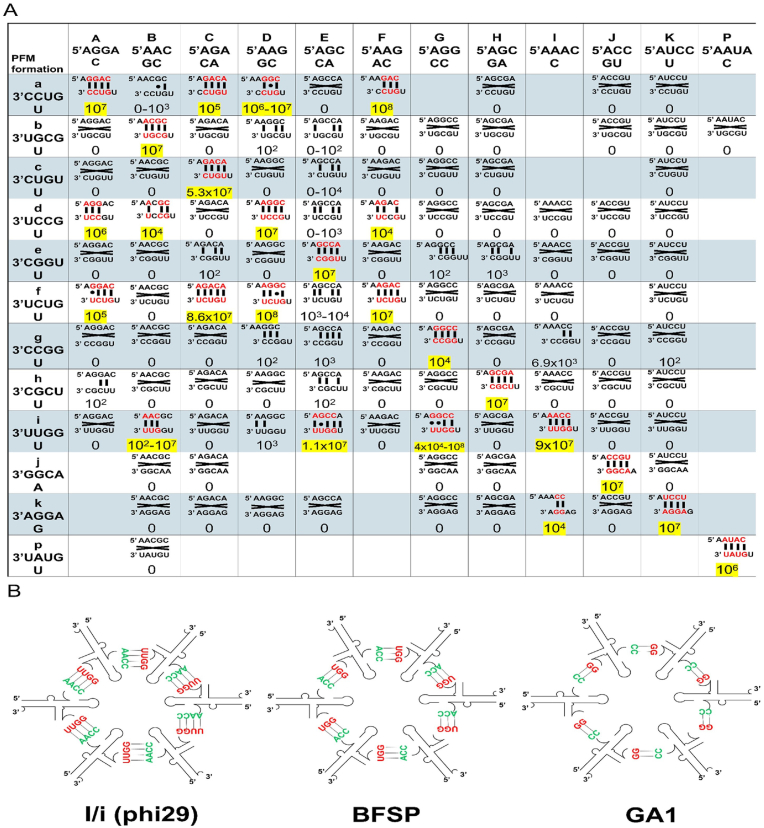
Verification of DNA packaging activity of phi29 pRNA with a variety of right-hand (RH) and left-hand loops (LH) mutations. **(A)** The RH and LH loops with complementary modification (paired as marked with two spots) were active (yellow color) due to forming a hexameric ring. Those RH and LH loops with unpaired mutation were inactive due to the disruption of paring as marked with a (X). The number is the activity of the pRNA as tested via DNA-packaging assay measured as viral plaque-forming unit (pfu). Due to the ultra-high number of PFU compared to the negative controls with zero, the significance of the difference and the reproducibility of results in the lab by different approaches, the experiment error in PFU was not calculated. **(B)** Base pairing patterns in the newly found pRNAs. The I/i pattern matches the phi29 family RH/LH interaction. One CC:GG pairing is sufficient for activity.

Phi29 pRNA with both LH and RH bulge mutations were tested individually in the phi29 DNA packaging system verified by different plaque-forming units (PFUs). As reported previously [32] and here, two base pairs are sufficient for biological function, but only if both are G/C pairs (GG/CC pair in Fig. 3A) since the melting temperature (*T*_*m*_) for the G/C pair is higher than that of the A/U pair. Experimental data revealed that a covariation of mutations is necessary. When the pairing sequence of LH bulge was mutated together with complementary RH bulge, the viral DNA packaging and assembly activity remained active. When the pairing was lost between the LH and RH bulges, the viral DNA packaging and assembly activity become inactive. Based on the criteria, the packaging activity of Phi29 family, BFSP1 family, vB_BceP_LY3, and the GA1 family pRNAs were identified (Fig. 3B). They all follow the pattern of a GG/CC pair with zero, one, or two additions of A/U pair in their LH and RH bulges (Fig. 3).

Single A/i pRNA or I/a pRNA with unpaired LH and RH loops remained inactive in phi29 genomic DNA packaging system when used individually (Fig. 3). When two inactive pRNAs (A/i and I/a) were mixed, A/a and I/i pair can form cross-pRNA complementary RH and LH loops. The DNA packaging and virus assembly activity were then verified. This indicates that the pRNAs form a ring with an even number, and the RH and the LH bulges are paired to form a stoichiometry of a multiple of 2. Similarly, when pRNAs with a pattern of A/i, I/j, or J/a were tested individually, none of the pRNAs had assembly activity (Fig. 3). When three pRNAs (A/I, I/j, and J/a) were mixed together [[32], [33], [34]], the viral assembly activity resumed. The result indicates the formation of a stoichiometry with a multiple of 3. These 2- and 3-unit symmetries indicate that the pRNAs form a hexameric functional complex only when the RH and the LH are paired to form a stoichiometry of a multiple of both 2 and 3. These data strongly support the finding that the pRNA ring on the phi29 DNA packaging motor is a hexameric ring [35].

### 2.3 The diversity of primary sequences of the pRNAs and the conservation of the 2D structure including the right-and left-hand loops, 3WJ, and critical guanidine in the ATP binding domains after evolution

**Fig. 4:**
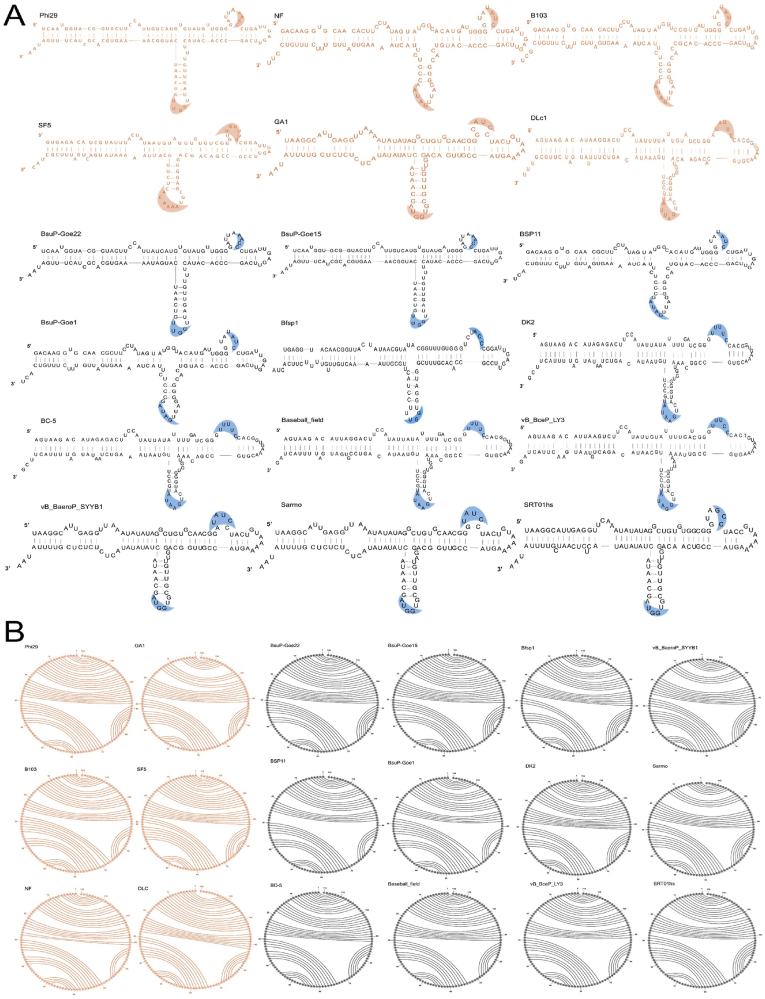
Secondary structure of 18 pRNA from different genomes of varieties of bacterial viruses. The published sequences are in orange, and the newly identified in this report are in black. **(A)** are results from M-fold results, and **(B)** is generated by RNAstructure Circular Plot Presentation.

Based on the primary sequences from the search, Mfold was used to obtain the 2D pictures based on the criteria of considering both the lowest ΔG energy and the existence of a clear three-way junction (3WJ) structure within the predicted secondary structure. The novel pRNA sequences were composed based on the 2D structure presentation of the dsDNA virus pRNA reported by Dwight Anderson and coworkers in 1990 [36] and further used and verified by Peixuan Guo and coworkers in 1999 [32] (Fig. 4A). The orange pRNA structures have been published by our lab and were used as the searching seeds, and the black pRNA structures are newly found pRNA structures (Fig. 4A). The left-and right-hand loops for the formation of the hexamer on the DNA packaging motor were identified in all 12 new pRNAs (Fig. 4A). Interestingly, it was found that mutation within one loop, for example, the left-hand loop, all led to a corresponding mutation within the right-hand loop. Even though the primary sequences of two pRNAs were relatively similar, the mutation of one interacting loop resulted in the corresponding mutation of the other loop to facilitate the base pairing of at least three or four nucleotides. This can be found in Fig. 4A, DLc1 vs. BC-5, DLc1 vs. Baseball_field, and BC-5 vs. vB_BceP_LY3. This corresponding mutation further confirms that pRNA can form pRNA complexes containing an even number of pRNAs, as reported before [28,32].

The secondary structures were further folded in a circular plot presentation via the RNA structure software developed by Matthews lab [37] (Fig. 4B). Similarly, the orange circular plot pRNAs were published by our lab and used as the searching seeds, and the black pRNA structures are newly found pRNA structures (Fig. 4B). The helix folding structures are clearly shown by the line connections within Fig. 4B. The conserved secondary structures between all the pRNAs are also clearly shown in Fig. 4B.

### 2.4 The presence of two other motor components in the same genomes confirms that these pRNAs are motor-gearing pRNAs

**Fig. 5:**
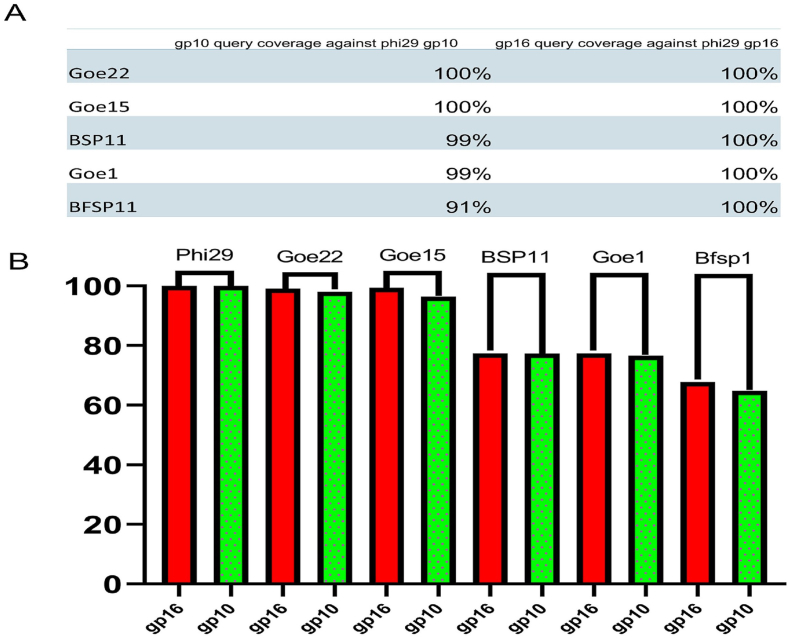
Verification of the authentication of five phi29-releted pRNA by assessing the amino acid sequence alignment score of the motor channel protein gp10 and the ATPase gp16. **(A)** is the query coverage and **(B)** is the percentage identity generate by protein BlastN.

The DNA packaging motors of dsDNA bacterial viruses contain a channel, an ATPase ring, and six pRNAs. Therefore, the validity of the 12 new pRNAs was further confirmed by the presence of two other key motor components, the channel protein gp10, and the ATPase gp16, in the same genomes where each pRNA originated. To verify that these pRNAs were indeed from the dsDNA bacterial virus, the existence of motor channel protein and the ATPase were searched by Protein Blast. It was confirmed that these pRNAs come from genomes that contain both the channel protein and the ATPase, supporting the conclusion that these RNAs are packaging motor pRNAs. The query coverage data comparing phi29 channel protein gp10 and ATPase gp16 with the corresponding functional proteins within the genomes of the newly found pRNAs are shown in Fig. 5A. The ATPase gp16 has a higher query coverage percentage compared to the motor channel gp10 protein, and the result indicates ATPase gp16 is more conservative compared to the motor channel gp10 protein on the primary sequence level.

Percentage identity was measured by comparing phi29 channel protein gp10 and ATPase gp16 with the corresponding functional proteins within the genomes of the newly found pRNAs (Fig. 5B). Percentage identity was calculated as the number of hits within the amino acid sequence of the channel protein or the ATPase divided by the total number of amino acids within channel protein or the ATPase. Fig. 5B compares the percentage identity of GOE22, GOE15, BSP11, GOE1, and BFSP11 channel proteins and ATPases. The protein level comparison further verifies that these two key protein components of the packaging motors are well conserved with high similarity in the amino acid sequence level; however, the primary pRNA sequence was not conserved as shown in Fig. 1, Fig. 2, and Table 1. The presence of two other motor components in the same genomes supports the conclusion that these pRNAs are indeed the motor-gearing components.

**Table 1:** Comparison of conserved rates in function and sequence of all pRNAs found by Infernal.

	pRNA	3-NT pair in L-/R-hand	3WJ structure	CCA relevant bulge	Conserved G to bind ATP
**Exist rate as function**	100 %	100 %	100 %	100 %	92 %
**Sequence Similarity**	0 %	0 %	0 %	0 %	92 %

### 2.5 The conservative 3D core structures of pRNAs are verified by the machine learning strategy

Different deep learning strategies, such as Alphafold3 [38] and trRosettaRNA [39], have been applied to predict the 3D structures of different biomolecules, including RNA [39,40]. Herein, Alphafold3 is used to predict the 3WJ core structure of all found pRNAs, and the conservative nature of the 3WJ core within most of the found pRNAs was identified.

**Fig. 6:**
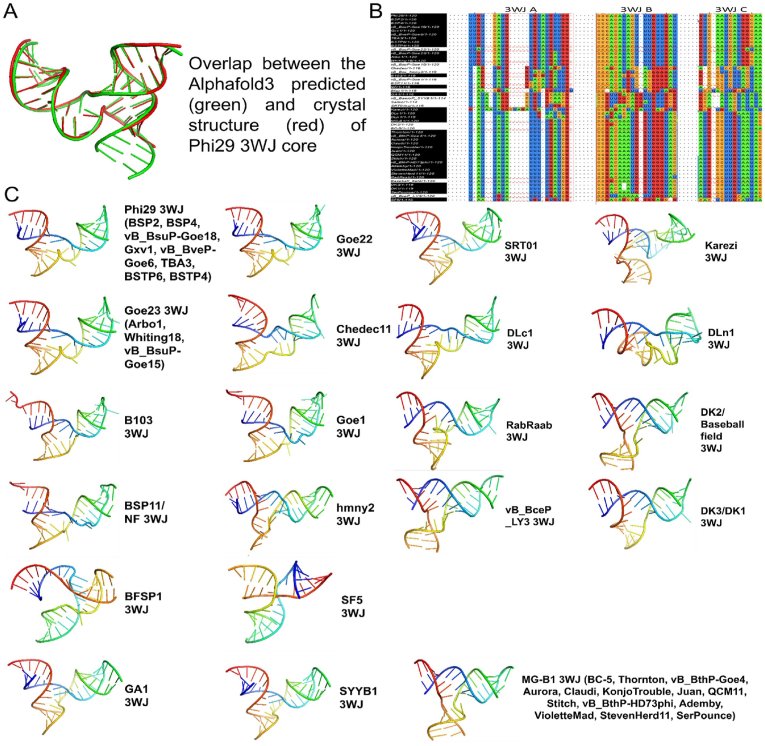
Conserved tertiary structure of pRNA 3WJ with unusually high T_M_ and low ΔG [[41], [42]]. **(A)** Overlap between Alphafold3 predicted structure (green) vs crystal structure (Zhang et al., 2013) of Phi29 3WJ Core (Red) presented by PyMol of the PDB. **(B)** Conserved primary sequence alignment of 3WJ core strands (3WJ-A, B, and C) extracted from Jalview data. **(C)** Similarity of AlphaFold predicted 3D structures of a variety of pRNAs.

The three-way junction (3WJ) of phi29 is the most stable structural core of pRNA with unusually high Tm and low ΔG [41,42]. Meanwhile, the crystal structure of the phi29 3WJ core has been reported [43]. Here, the structure conservation of the 3WJ was analyzed. The AlphaFold was used to predict the 3WJ 3D structure. To evaluate the feasibility of using the Alphafold3 for pRNA 3WJ folding, a benchmark comparison was made by comparing the Alphafold3 predicted phi29 3WJ core with the crystal structure of the phi29 3WJ core (PDB entry id: 4KZ2) in the database. As shown in Fig. 6A, a complete overlap was observed by comparing the Alphafold3 predicted phi29 3WJ core and the crystal structure PDB data. The consistency of the two 3D structures suggests the reliability of AlphaFold in 3WJ 3D structure prediction. Primary sequence alignment (Fig. 6B) revealed that the nucleotides of the 3WJ hold relatively fewer mutations, suggesting that the stable 3D structure has limited the mutation during evolution. 3D structure prediction by AlphaFold revealed that the 3D structure is well conserved. Among the 50 found pRNA structures, 49 of them can form clear 3WJ structures when the core sequence is given to Alphafold3, except for the Sarmo 3WJ core. Every 3WJ structure shows very similar 3D structures (Fig. 6C), indicating the strong conservation during the evolution due to the high Tm of the 3WJ structure.

### 2.6 Phylogenetic Analysis of pRNAs

**Fig. 7:**
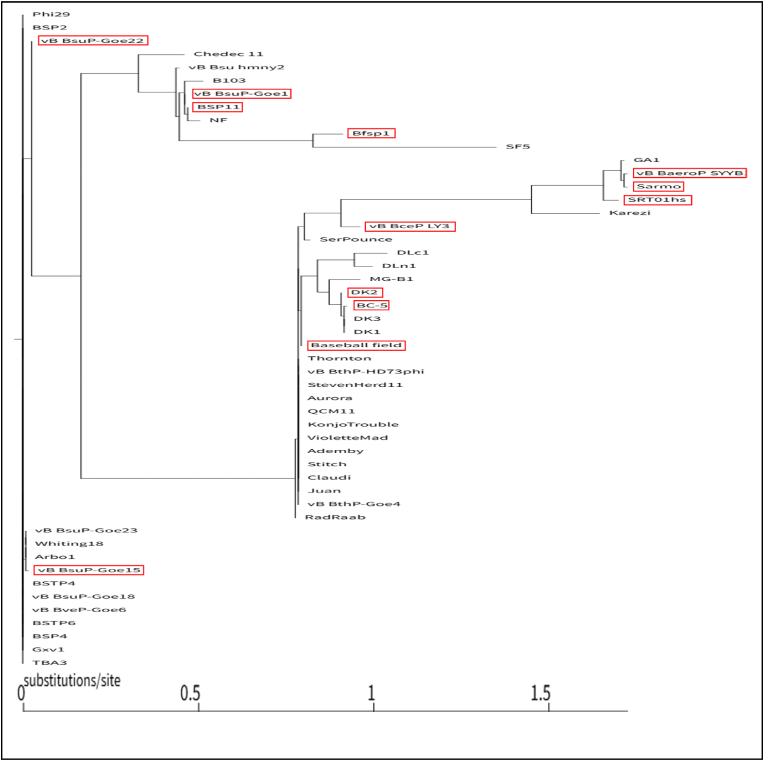
Phylogenetic Analysis of all 50 RNAs shows the evolutionary branching relationships between all found pRNAs. 12 newly found pRNAs are shown as red labeled.

Phylogenetic analysis of pRNAs in this study was performed to understand the evolutionary relationship between different viruses. The 50 pRNAs in this study were analyzed phylogenetically via IQ-Tree. The horizontal distance determines their substitutions/site number between individual pRNA as each node. The higher number of the substitutions/site indicates a larger evolutionary distance. A clear evolutionary branching relationship among all 50 found pRNAs was determined (Fig. 7). The largest Substitutions/site value difference was between Sarmo and Phi29, calculated as around 1.7 (Table 2). This relatively high number indicates a high substitution rate for these nucleotides during evolution. However, as shown in Fig. 4, Fig. 6, a similar pRNA 2D structure (Fig. 4) and 3WJ 3D structure (Fig. 6) are shared between Phi29 and Sarmo. The conserved 2D and 3D structures are due to the evolutionary pressures acting onto the viruses.

**Table 2:** Substitutions/site calculated based on Phylogenetic Tree.

Node	Substitutions/site
BSP2	5.53E-06
Arbo1	0.008428201
vB_BsuP-Goe23	0.008428201
vB_BsuP-Goe15	0.016821869
vB_BsuP-Goe22	0.025465314
vB_Bsu_hmny2	0.446704927
Chedec_11	0.460794687
vB_BsuP-Goe1	0.462088788
BSP11	0.470620508
NF	0.505390375
B103	0.514437244
RadRaab	0.777988217
Thornton	0.786363866
vB_BthP-Goe4	0.786366631
Baseball_field	0.794721825
SerPounce	0.820179772
DK2	0.908847473
Bfsp1	0.913337737
DK3	0.91722351
DK1	0.91722351
BC-5	0.925633102
vB_BceP_LY3	0.962381086
MG-B1	0.962581741
DLn1	0.998228907
DLc1	1.040174574
SF5	1.351588569
Karezi	1.646120999
SRT01hs	1.700520042
GA1	1.716587057
vB_BaeroP_SYYB1	1.725374392
Sarmo	1.725406377

## 3 Methods

### 3.1 RNA primary structure-based searching using BlastN

Standard Nucleotide Blast was used to search against multiple seed pRNAs using NIH online service (https://blast.ncbi.nlm.nih.gov/Blast.cgi?PROGRAM=blastn&PAGE_TYPE=BlastSearch&LINK_LOC=blasthome). The program used was blastn, and the database used was Core nucleotide database (core_nt) among standard databases. Algorithm parameters were left unchanged. In brief, the Max target sequences was set to 100, short queries box was checked, expect threshold was set to 0.05, word size was set to 11, Max matches in a query range were set to 0. Match/Mismatch Scores were set to 2,-3. Gap Costs were set to existence: 5 Extension: 2. Low complexity regions and Mask for lookup table only boxes were checked. The rest parameters were left blank or unchecked. Phi29 pRNA and DLc1 pRNA sequences were found within the complete genomes of phi29 (NC_011048.1) and DLc1 (NC_055908.1). GA1 pRNA was reported in the paper referred here [28]. The results of searching for phi29, DLc1, and GA1 were downloaded from the website and summarized into the table.

### 3.2 RNA secondary structure-based searching using infernal package

Infernal 1.1.5 with MacOSX/Intel binaries was downloaded from http://eddylab.org/infernal/. The virus complete genome database in fasta format was downloaded from NCBI. Phi29 pRNA and DLc1 pRNA sequences were found within the complete genomes of phi29 (NC_011048.1) and DLc1 (NC_055908.1). GA1 pRNA was reported in the paper referred here [28]. CMSearch function was used to search pRNAs of phi29 and DLc1 against the complete genome database with all default settings of the CMSearch function. The local alignment algorithm was used. All hits with an E-value ≤ 10 were reported.

### 3.3 RNA sequence alignment of hit pRNAs and newly discovered pRNAs

Altogether, 50 pRNA hits and pRNA seeds (Phi29, GA1, NF, B103, SF5, and DLc1) were aligned using the Tcoffee with Defaults and presented in a nucleotide-colored format using Jalview.

### 3.4 RNA activity assay with the highly sensitive phi29 assembly system and changes in the LH and RH bulges

The activity of pRNAs with different pairs of mutation in the LH and RH bulges was assayed based on the previously published paper [32] with modifications. In brief, the activity of pRNAs was evaluated using the highly sensitive phi29 *in vitro* assembly system. The purification of procapsids, gp16, DNA-gp3, and neck and tail protein extracts followed established protocols. The activity of each mutated pRNA was quantified based on the number of plaque-forming units per milliliter when 50 ng of pRNAs were used in the viral assembly assay, confirming that pRNA functions solely in the DNA packaging step. pRNAs producing fewer plaque-forming units (10^4^) than the wild-type control (10^7^) were considered to have reduced activity.

### 3.5 RNA 2D structure formation and base pair searching to find the two eligible bulges with base pairing existed

The secondary structure folding prediction was generated in a pdf format via the Mfold webserver (https://www.unafold.org/mfold/applications/rna-folding-form.php) using linear as the RNA sequence, 37° settled as the folding temperature, 1M NaCl settled as the ionic conditions, 15 settled for percent suboptimality number, 50 settled for the an upper bound on the number of computed folding, default settled as the window parameter, 30 settled for maximum interior/bulge loop size, 30 settled for maximum asymmetry of an interior/bulge loop, and no limit settled for the maximum distance between paired bases. Within potential multiple folded structures, a structure with a combination of a lower ΔG, a 3WJ core, and the existence of the LH/RH bulges was chosen for the subsequent analysis. The potential base pair relationship between two eligible bulges was determined by the string searching algorithm between left- and right-hand bulges. The structure was first drawn following the consensus of the referred paper [36]. The format was further converted into the Stockholm format for Structure Editor to generate the circular RNA format for presentation. Structure editor was downloaded from Mathews lab (https://rna.urmc.rochester.edu/RNAstructureDownloadArchive.php).

### 3.6 Critical packing protein identification and protein blast search

Both connector and ATPase protein were searched within the CDS area of the complete genome of the virus with found pRNA. The connector protein may have other names such as upper connector, portal protein, or gp10, and the ATPase protein may have names such as DNA packaging protein, DNA encapsulation protein, or gp16. Protein Blast was used to search for phi29 gp10 and gp16 against the whole protein database. The phi29 gp10 used as the blastp seed was with a RefSeq Accession of YP_002004539.1, and the phi29 gp16 used as the blastp seed was with a RefSeq Accession of YP_002004545.1. The database used was Non-redundant protein sequences (nr). The algorithm used was blastp. Max target sequences used were 100. Short queries box was checked. Expect threshold used was 0.05. Word size used was 5. Max matches in a query range used were 0. Matrix used was BLOSUM62. Gap costs used were Existence: 11 Extension: 1. Compositional adjustments used were conditional compositional score matrix adjustment. The rest parameters were left blank or unchecked. The found proteins that share the similarity with either phi29 gp10 or phi29 gp16 were calculated for both query coverage and percentage identity. Query coverage measured the percentage of queried phi29 gp10 or phi29 gp16 that was included in the matched gp10 or gp16. Percentage identity was calculated by found hit-protein gp10 or gp16 amino acid sequence/phi29 gp10 or gp16 protein amino acid sequence.

### 3.7 RNA tertiary structures prediction with Alphafold3

RNA 3WJ structure was identified via the Mfold with the same protocol described previously. The three separated strands that made up the 3WJ core were extracted and folded by the Alphafold3 online server (https://alphafoldserver.com/) using auto seeds provided by Alphafold3. A predicted model with a pTM number higher than 0.35 was accepted and the model was presented within PyMol.

### 3.8 Phylogenetic Analysis of all found pRNAs using IQ-tree

The aligned structures of 50 pRNAs were determined using Jalview as described above. The online version of the IQ tree (https://www.hiv.lanl.gov/content/sequence/IQTREE/iqtree.html) was used to draw the phylogenetic tree. The sequence type was set to nucleotides, and the model was set as GTR. Create site rates file box was checked. The state frequency was set to Estimated by Maximum Likelihood. The root tree was set to None. The tree type was set to Unrooted and Phylogram. The rest parameters were left blank or unchecked. The phylogenetic tree was automatically generated by IQ-Tree with a substitutions/site scale bar at the bottom. The generated Tree in newick format was used to calculate the distance between phi29 pRNA node and all the other nodes.

### 3.9 Computation of the existence rate and similarity on the sequence rate

Multiple different parameters were considered and calculated for both the existence rate and similarity of the sequence rate. The existence rate measurement was determined by whether the columns of residues of the structure of interest within the aligned Jalview data were empty or not, and the similarity in the sequence rate was determined by whether the residues of the structure of interests within the aligned Jalview data were the same between different species. For example, the existence rate of the three-base pairing of the left and right-hand loops was checked by the Bulge1 line and Bulge3 residues of the Jalview aligned data. All bulge1 and bulge3 residue columns were not void. The similarity in the sequence rate was measured by checking the Jalview aligned section of bulge1 and bulge3, and the numbers were zero because neither bulge1 nor bulge3 sequences match exactly between different species. The calculation was further applied to the 3WJ structure, secondary bulge, critical G for the ATP binding, and overall pRNA.

## 4 Discussion

The fundamental characteristic of life is movement. Living systems are maintained by a set of nanomachines that can replicate and adapt to perform complex functions. Biological ATPase motors are ubiquitous nanoscale machines in biological systems, participating in many microscopic movements such as cell mitosis [44], macromolecular transport [45], DNA and RNA replication [46], transcription of biological activities [47], DNA repair [48], and genome packaging [49,50].

ATPases are a class of enzymes that catalyze the breakdown of adenosine triphosphate (ATP) into adenosine diphosphate (ADP) and free phosphate ions [51]. They are the main energy source for the mechanical work of biological engines [51]. For the ATPases biomotors with multiple subunits, the conversion from chemical to mechanical energy usually occurs sequentially between subunits, accompanied by conformational changes in the motors [52,53]. ATP is the energy source that drives the motion. The delicate and complex structure of many ATPase motors has inspired the design of biomimetic machines for nanotechnology applications [54,55]. These ATPases adopt a multi-subunit ring structure to generate force through the synergy of multiple modules [54,55]. Most motors employ a multi-subunit ring structure that hydrolyzes ATP to generate force [54]. It is noteworthy how these motors control the direction of motion and regulate the sequential actions of multiple subunits. Revolving [56] and rotary motion motors [57] have been reported, and transmission substrate-based motion mechanisms have been further issued [58]. However, for phi29's DNA-packaging motor, a series of debates about whether the motors are fivefold [59] or sixfold [28] has been ongoing.

In the present study, we used secondary structure-based homology searches to identify 12 candidates as novel pRNAs from different species that gear an ATPase based biomotor. To bind to the DNA-packaging motor, pRNAs multimerize via hand-in-hand interactions of the complementary right-hand and left-hand loops. This fact corroborates the identities of our novel pRNAs since we observed that variance in one binding region (i.e. the right hand) was associated with a complementary variant in the other binding region (i.e. the left hand). This phenomenon supports the idea that these novel pRNAs must multimerize via hand-in-hand interactions in order to gear the DNA-packaging motor; if a mutation only affected one binding region and not the other, the DNA-packaging motor would be rendered nonfunctional, and therefore, this mutation would have not been evolutionarily selected.

Furthermore, we identified homologues to the phi29 ATPase gp16 and connector gp10 proteins, both of which are necessary for viral dsDNA-packaging, in the genomes of the viruses with novel pRNAs. This further supports that these pRNAs function as a part of a DNA-packaging motor. Additionally, this is notable because the phi29 DNA-packaging motor requires that all six pRNAs be functional; if a single pRNA monomer is nonfunctional, the entire system breaks [60]. This phenomenon would prompt selection against mutations that result in an inability to participate in hand-in-hand interactions.

Experimental data have proposed and partially support the theory that RNA is the origin of life [61]. The recent finding that RNAs are motile and reformative organisms supports the “RNA world” hypothesis [6,62,63]. Previously, we reported an antique pRNA of *Bacillus* virus phi29 [27]. Phi29 can infect spore-forming *Bacillus subtilis*, hidden within the soil for an undefined long period of time without being subjected to evolution due to the protection of the spore and lack of replication. Moreover, many other pRNAs with a similar secondary structure to that of phi29 were found in this research and other published research, and the existence of many packaging motor-gearing pRNAs further indicates these conserved pRNA structures are a necessity to gear the packaging motor during the evolution of viruses. The widespread existence of pRNAs indicates that pRNA may have existed among the earliest viruses on Earth and remain after an estimated 3 billion years of evolution [64]. The conservative evolving nature of the phi29 bacteria virus and the important function of non-coding packaging RNA within bacteria viruses all indicate that RNA performs an important role in biological function in early life, supporting the “RNA world” hypothesis.

## 5 Conclusion

The RNA world hypothesis may explain the origin of life, and evidence for the important functionality of RNA among early life will help to solidify the RNA world hypothesis. The importance of pRNA within different species, and the fact that phi29 and its family viruses are ancient species, all support the role of RNA in early life and the hypothesis of the RNA world. The primary sequences of the pRNAs are different, while the secondary and tertiary structures are similar. The evolution is minimal at the secondary structure (2°) level and tertiary (3°) level, while it is greater at the primary structure (1°) level. The evolution of RNA is analogous to the fact that genes change greatly, and proteins remain unchanged. Finding more pRNA analogs in nature could help to better prove the RNA world hypothesis and the important biological function of RNA in early life. Studying the evolution of ncRNA can provide a unique theoretical basis for a specific targeting treatment and specific disease diagnosis *in vivo* using RNA nanotechnology.

## CRediT authorship contribution statement

**Kai Jin:** Writing – original draft, Visualization, Validation, Software, Methodology, Investigation, Formal analysis, Data curation. **Margaret Bohmer:** Writing – original draft, Visualization, Validation, Investigation. **Peixuan Guo:** Writing – original draft, Visualization, Validation, Supervision, Software, Resources, Project administration, Methodology, Funding acquisition, Conceptualization.

## Data availability statement

Raw data and materials presented here are available upon written request to P.G.

## Declaration of competing interest

P.G. is the cofounder of ExonanoRNA, LLC and the consultant of RNA NanoBiotics.
