# Supplementary material for: Illustration of the variable 1D sequences but conserved 2D and 3D structures of different ncRNA nanostructures for tracking the evolution and origin of organisms
Source: Noncoding RNA Res. 2025 Sep 30;16:156–66. doi: 10.1016/j.ncrna.2025.09.003 (PMC12670572; doi:10.1016/j.ncrna.2025.09.003)
Supplement: Multimedia component 1 [file mmc1.docx]

Table 1. Comparison of conserved rates in function and sequence of all pRNAs found by Infernal

|  | **pRNA** | **3-NT pair in L-/R-hand** | **3WJ**  **structure** | **CCA**  **relevant budge** | **Conserved G to bind ATP** |
| --- | --- | --- | --- | --- | --- |
| **Exist rate as function** | 100% | 100% | 100% | 100% | 92% |
| **Sequence Similarity** | 0% | 0% | 0% | 0% | 92% |

**Table 2. Substitutions/site calculated based on Phylogenetic Tree**

| Node | Substitutions/site |
| --- | --- |
| BSP2 | 5.53E-06 |
| Arbo1 | 0.008428201 |
| vB_BsuP-Goe23 | 0.008428201 |
| vB_BsuP-Goe15 | 0.016821869 |
| vB_BsuP-Goe22 | 0.025465314 |
| vB_Bsu_hmny2 | 0.446704927 |
| Chedec_11 | 0.460794687 |
| vB_BsuP-Goe1 | 0.462088788 |
| BSP11 | 0.470620508 |
| NF | 0.505390375 |
| B103 | 0.514437244 |
| RadRaab | 0.777988217 |
| Thornton | 0.786363866 |
| vB_BthP-Goe4 | 0.786366631 |
| Baseball_field | 0.794721825 |
| SerPounce | 0.820179772 |
| DK2 | 0.908847473 |
| Bfsp1 | 0.913337737 |
| DK3 | 0.91722351 |
| DK1 | 0.91722351 |
| BC-5 | 0.925633102 |
| vB_BceP_LY3 | 0.962381086 |
| MG-B1 | 0.962581741 |
| DLn1 | 0.998228907 |
| DLc1 | 1.040174574 |
| SF5 | 1.351588569 |
| Karezi | 1.646120999 |
| SRT01hs | 1.700520042 |
| GA1 | 1.716587057 |
| vB_BaeroP_SYYB1 | 1.725374392 |
| Sarmo | 1.725406377 |
